# Post-stroke cognitive impairment and synaptic plasticity: A review about the mechanisms and Chinese herbal drugs strategies

**DOI:** 10.3389/fnins.2023.1123817

**Published:** 2023-03-01

**Authors:** Xiansu Chi, Liuding Wang, Hongxi Liu, Yunling Zhang, Wei Shen

**Affiliations:** ^1^Xiyuan Hospital, China Academy of Chinese Medical Sciences, Beijing, China; ^2^Graduate School, Beijing University of Chinese Medicine, Beijing, China

**Keywords:** post-stroke cognitive impairment, mechanisms, synaptic plasticity, structural synaptic plasticity, functional synaptic plasticity, Chinese herbal drugs

## Abstract

Post-stroke cognitive impairment, is a major complication of stroke, characterized by cognitive dysfunction, which directly affects the quality of life. Post-stroke cognitive impairment highlights the causal relationship between stroke and cognitive impairment. The pathological damage of stroke, including the increased release of excitatory amino acids, oxidative stress, inflammatory responses, apoptosis, changed neurotrophic factor levels and gene expression, influence synaptic plasticity. Synaptic plasticity refers to the activity-dependent changes in the strength of synaptic connections and efficiency of synaptic transmission at pre-existing synapses and can be divided into structural synaptic plasticity and functional synaptic plasticity. Changes in synaptic plasticity have been proven to play important roles in the occurrence and treatment of post-stroke cognitive impairment. Evidence has indicated that Chinese herbal drugs have effect of treating post-stroke cognitive impairment. In this review, we overview the influence of pathological damage of stroke on synaptic plasticity, analyze the changes of synaptic plasticity in post-stroke cognitive impairment, and summarize the commonly used Chinese herbal drugs whose active ingredient or extracts can regulate synaptic plasticity. This review will summarize the relationship between post-stroke cognitive impairment and synaptic plasticity, provide new ideas for future exploration of the mechanism of post-stroke cognitive impairment, compile evidence of applying Chinese herbal drugs to treat post-stroke cognitive impairment and lay a foundation for the development of novel formulas for treating post-stroke cognitive impairment.

## 1. Introduction

Post-stroke cognitive impairment (PSCI), one of the major complications after stroke, occurs in the 3 to 6 months after stroke onset and is characterized by impairment of cognitive function (Mijajlović et al., 2017; Rost et al., 2022). Nowadays, about one-third of stroke survivors manifest considerable cognitive impairment within a few months after stroke (Gorelick et al., 2011). Based on the analysis of the cognitive status of 3,146 patients of stroke (97%) or transient ischemic attack (3%) from eight countries, Lo et al. found that within 2 to 6 months after stroke, 44% of patients were found overall cognitive impairment and 30–35% of stroke survivors had the impairment in a single cognitive domain (Lo et al., 2019). PSCI can affect quality of life, prevent patients from participating in social activities, and increase the burden on families (Zhang and Bi, 2020).

**FIGURE 1:**
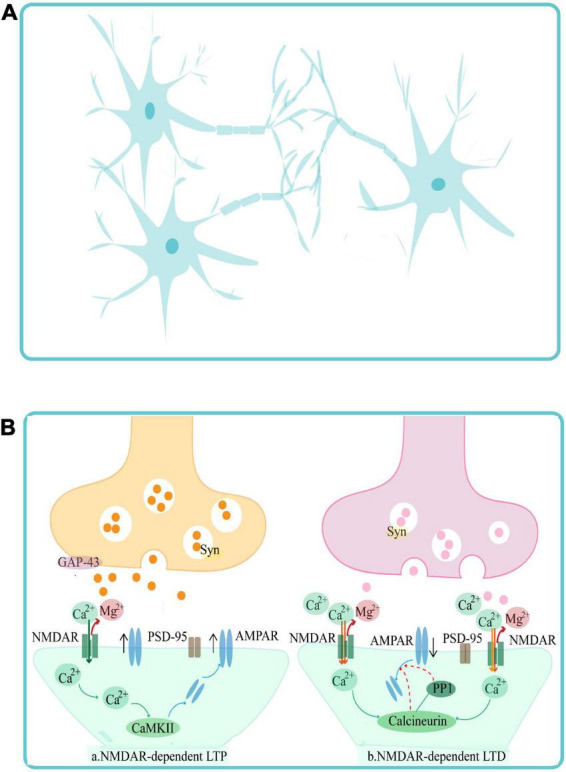
Synaptic plasticity can be categorized into structural synaptic plasticity **(A)** and functional synaptic plasticity **(B)**. Structural synaptic plasticity panel **(A)** refers to the number, density, distribution of synapses, and highlights the strength of synaptic connections. Functional synaptic plasticity panel **(B)** refers to the efficiency of synaptic transmission, and includes NMDAR-dependent LTP and NMDAR-dependent LTD. GAP-43, PSD-95, Syn, CaMKII, NMDAR, and AMPAR are synaptic plasticity-related proteins, which play important roles in mediating changes in synaptic morphology, structure, and density and in the function of inter-synaptic information transfer.

PSCI highlights the causal relationship between stroke and cognitive impairment. Thus, understanding the complex pathological mechanism between stroke events and cognitive disorders is essential for the development of targeted intervention and treatment strategies. Evidence shows that stroke induces synaptic plasticity impairment, which may be a potential mechanism for PSCI (Joy and Carmichael, 2021). Synaptic plasticity refers to the activity-dependent changes in the strength of synaptic connections and efficiency of synaptic transmission at pre-existing synapses (Mateos-Aparicio and Rodríguez-Moreno, 2020). It can be divided into structural synaptic plasticity and functional synaptic plasticity (Figure 1). Structural synaptic plasticity refers to adaptive changes in the synaptic ultrastructure such as number, density, and distribution of synapses, highlighting the strength of synaptic connections (Sadigh-Eteghad et al., 2018). Functional synaptic plasticity refers to the efficacy of synaptic transmission, including long-term potentiation (LTP) and long-term depression (LTD). Synaptic plasticity is closely related to the recovery and improvement of cognitive impairment, the increase in the strength of synaptic connections and efficiency of synaptic transmission directly upregulate the processing and storage of information within the central nervous system, thus improving the cognitive function (Raven et al., 2018).

Currently, there is limited evidence to support the effectiveness of therapeutic strategies in the treatment of PCSI. Considering their overlap with the neuropathological mechanisms of PSCI (Mok et al., 2017), the choice of treatments for PCSI currently relies on the evidence of the drugs used in the treatment of vascular cognitive impairment, vascular dementia, and Alzheimer’s disease (Wang et al., 2021). In terms of Western medicine treatment options, donepezil, an acetylcholinesterase inhibitor, is recommended for improving cognitive function and activities of daily living in PSCI patients, with the character of safe and well-tolerated. Meanwhile, galantamine, the cholinesterase inhibitor, is potentially effective for PSCI but is less safe and less tolerated (Whyte et al., 2008). Additionally, the antioxidant drug dimethyl fumarate has been demonstrated to improve learning and memory function in a rat model of PSCI, possibly by reducing apoptosis and autophagy and exerting anti-oxidative effects *via* the Nrf2-ARE (Hou et al., 2020), however, reported side effects include a 30% drop in lymphocyte count after administration and an increased risk of infection, which cannot be ignored (Berkovich and Weiner, 2015). Thus, extensive clinical and mechanistic studies are still needed. Non-pharmacological therapies are also the effective strategy for PSCI, such as enriched environments, physical activity, lifestyle interventions, and acupuncture (Liu et al., 2021; Wang et al., 2021), however, large-scale controlled trials are still lacking. Furthermore, Chinese herbal drugs, as a class of natural medicinal products based on the theory of traditional Chinese medicine, are widely used in Asia and play an important role in improving cognitive function in patients with PSCI. In our previous research, python language data was used to optimize and integrate the medication rules of Chinese herbal drugs in the clinical treatment of PSCI, and 214 Chinese herbal drugs with a total frequency of 1,685 times were found (Shen et al., 2022). However, the mechanism of these Chinese herbal drugs in treating PSCI still needs to be further explored and summarized.

Nowadays, strategies that regulate synaptic plasticity have been proven to play an important role in improving cognitive impairment (Bordet et al., 2017), but the specific mechanisms are still unclear. In this review, we overview the influence of pathological damage of stroke on synaptic plasticity, analyze the changes of synaptic plasticity in PSCI, and summarize the applications and the underlying mechanisms of Chinese herbal drugs in the treatment of PSCI from the perspective of synaptic plasticity. This review will provide new ideas for future exploration of the mechanism of PSCI, and compile evidence of applying Chinese herbal drugs to treat PSCI.

## 2. Pathological damage of stroke influence synaptic plasticity

**FIGURE 2:**
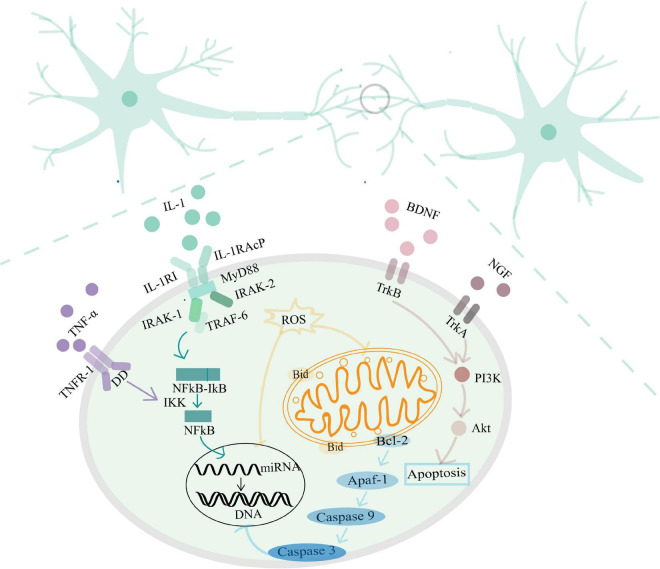
The mechanism of the pathological damage of stroke influencing synaptic plasticity.

Stroke is an important cause of PSCI and accompanied with a series of pathological damage (Joy and Carmichael, 2021). During the hyperacute phase of stroke, inadequate perfusion limits the supply of oxygen and glucose. Subsequently, ATP deficiency leads to abnormal ion pump operation, triggering the depolarization of neurons and glial cells, and abnormal extracellular accumulation of glutamate resulting in excitotoxicity (Karaszewski et al., 2009; Castillo et al., 2016). Excessive glutamate release also leads to the excessive activation of *N*-methyl-D-aspartic acid receptors (NMDAR), increased the inward flow and overload of deficiency calcium (Ca^2+^), which trigger mitochondrial damage, and a series of oxidative stress responses (Chan, 2001; Iadecola and Anrather, 2011), which are accompanied by inflammatory cytokine release (Taylor and Sansing, 2013). The acute phase in the week following stroke is characterized by neuronal apoptosis and further activation of immune responses (Anrather and Iadecola, 2016). In the subacute phase, alterations in transcriptional growth factor activity and gene expression can impact synaptic plasticity. Approximately 500 different neuronal genes in the peri-infarct region regulate nerve growth factor expression and cytoskeletal rearrangements (Li et al., 2010), influencing the axonal growth and synapse formation. This alteration in plasticity diminishes when entering into the chronic phase and the reduced strength and transmission efficiency of synaptic connections continuously affects brain function (Joy and Carmichael, 2021). A series of pathological damage of stroke, including increased release of excitatory amino acids, oxidative stress, inflammatory responses, apoptosis, changed neurotrophic factor levels and gene expression influence synaptic plasticity (Figure 2), and ultimately participate in the development of PSCI. The specific mechanism is as follows.

### 2.1. Increased release of excitatory amino acid and synaptic plasticity

Glutamate has complex functions in excitatory neurotransmission; in addition to its role as a neurotransmitter, glutamate regulates neuronal survival, neurite growth, and synaptogenesis (Mattson, 2008). However, disturbed clearance of glutamate in the synaptic cleft results in synaptic dysfunction (Postnikova et al., 2021). Glutamate excitotoxicity triggers excessive activation of CDK5, which increases Ca^2+^ influx in neuronal cells, it will promote dendritic retraction, spine loss, increased endoplasmic reticulum mitochondrial Ca^2+^, and ultimately neuronal death (Toro-Fernández et al., 2021). These changes manifest clinically as a decline in memory, cognition, and general functioning (McGrath et al., 2022). Moreover, disturbed glutamate system after cerebral ischemia injury, has been indicated to participate in the development of cognitive impairment (Nie and Yang, 2017).

### 2.2. Oxidative stress and synaptic plasticity

Mitochondria maintain energy supplement to neuronal activity, and play an important role in synaptic plasticity and neurotransmitter synthesis. Because of the high oxygen consumption with restricted antioxidant mechanisms, neurons are vulnerable to oxidative stress, thus, protecting the integrity and survival of neurons from oxidative stress damage is vital (Cenini et al., 2019). Chronic oxidative stress can impact synaptic plasticity in various ways, for example, through the loss of dendritic spines, neurons, or brain repair mechanisms, resulting in the loss of synaptic connections and the ability to process information. Moreover, oxidative stress can also affect gene expression (Bello-Medina et al., 2019). The structure and function of neurons are very sensitive to pathological and physiological changes, and oxidative stress responses and decreased antioxidant capacity can significantly impair learning and memory function by reducing the production of new neurons and altering the structure of dendrites in the hippocampus (Huang et al., 2015).

### 2.3. Inflammatory response and synaptic plasticity

Homeostatic regulation of synaptic function is the key principle of the nervous system, and molecules associated with inflammatory responses are critical in regulating synaptic plasticity. Cytokines are released by resident myeloid cells to maintain synaptic plasticity. When cytokines release is disordered, neuroinflammation can be triggered which negatively influences the brain networks associated with learning and cognition, contributing to neurodegeneration (Rizzo et al., 2018). Pro-inflammatory cytokines, such as interleukin (IL)–1, and tumor necrosis factor (TNF-α), induce the activation of transcription factor NF-κB and initiate the expression of downstream genes related to inflammation, thus inducing an inflammatory response. These above inflammatory response activates the microglia and astrocytes, can down-regulate synaptic plasticity and synaptic scaling in several core brain areas, such as the cortex, striatum, and hippocampus (Yirmiya and Goshen, 2011; Yang et al., 2013). Evidence indicates that low doses of IL-1 facilitate learning processes, whereas mice with genetic impairments or pharmacological blockade of IL-1 signaling and downregulated levels of IL-1 have decreased cognitive and memory functions (Yirmiya and Goshen, 2011). Likewise, high level of TNF-α impair synaptic plasticity in pyramidal neurons by regulating signaling pathways which are modulated by intracellular Ca^2+^ stores and synaptopodin (Maggio and Vlachos, 2018). The inflammatory response is the main cause of secondary injury after ischemic stroke.

### 2.4. Apoptosis and synaptic plasticity

Protection from cerebral ischemia-reperfusion injury can be achieved by reducing the apoptosis rate of ischemic and hypoxic brain cells, regulating the plasticity of synaptic structures by inhibiting the activity of neuronal apoptosis-related factors, and promoting neuronal regeneration (Xia and Lin, 2021). The apoptosis activated at the end of the synapse or in the synapses, leads to the local functional and morphological changes of the synapse, and even spreads to the cell body, ultimately resulting in neuronal death. Evidence indicates that the activation of the caspase family at the end of the synapse is used to respond various stimuli (Mattson and Duan, 1999). Caspases have important functions in apoptosis, and inflammation. After activation by B-cell lymphoma 2 (Bcl-2), apoptotic protease activating factor 1 (Apaf-1) regulates the level of caspase-3 and caspase-9, resulting in the promotion of apoptosis and even affects DNA expression. Restricted and localized caspase activation within neurons also participates in axon and dendrite pruning, neurite outgrowth, and dendrite branch formation, and LTD (Hollville and Deshmukh, 2018). Finally, the low strength and efficiency of synaptic connections induced by caspase manifests as cognitive impairment.

### 2.5. Neurotrophic factors and synaptic plasticity

A growing number of publications have focused on the relationship between the expression of neurotrophic factors such as brain-derived neurotrophic factor (BDNF) and the nerve growth factor (NGF) and synaptic plasticity. Expression of BDNF and its receptor tropomyosin-associated kinase B (TrkB) supports the morphology, differentiation and regeneration of neurons, axonal growth, and the functions of synapses after neurological injury (Griesbach et al., 2004). BDNF is the main activity-dependent neurotrophin on which active neuronal organization relies. In a synapse-specific manner, BDNF balances the effects of excitatory and inhibitory transmission (Spedding and Gressens, 2008). NGF and its receptors (P75 and TrkA) can regulate cholinergic neuronal markers, facilitate LTP induction in structural synaptic plasticity, increase neurite outgrowth, and promote synaptic plasticity (Conner et al., 2009). Neurotrophic factors regulate synaptic connections and dendritic morphology, thereby influencing synaptic plasticity and cognitive function.

### 2.6. Gene expression and synaptic plasticity

The development of synapses and the transmission of synaptic signals are regulated by gene expression, and neuronal genes in the peri-infarct region will influence synaptic plasticity. miRNAs regulate gene expression and interact with 3’-untranslated regions of mRNAs to maintain mRNA stability and promote mRNA translation, thereby regulating synaptic activity, as well as protein synthesis and expression (Hu and Li, 2017). Although the expression of synaptic miRNAs is region-specific, they are core ingredients of dendrites, axons, and dendritic spines and can be detected in synaptoneurosomes (enriched because of dendritic spines), synaptosomes (consisting of axon terminals and adherent postsynaptic densities), and postsynaptic densities (Lugli et al., 2008). Different miRNAs play distinct roles in regulating synaptic plasticity. The inactivation of *Dicer1* in excitatory hippocampal neurons leads to increases in dendritic spine length, neural excitability, and post-tetanic potentiation (Fiorenza et al., 2016). The knockout of Ncoa3 is accompanied by a reduction in the volume of dendritic spines (Störchel et al., 2015). Substantial evidence indicates that miR-125b, miR-223, miR-137, and miR-146a-5p modulate postsynaptic responses by controlling the abundance of postsynaptic glutamate receptors. Among these, miR-125b controls the expression of the NMDAR subunit GluN2A (Edbauer et al., 2010), whereas miR-223 controls the NMDAR subunit GluN2B and the α-amino-3-hydroxy-5-methyl–4-isoxazole propionic acid receptor (AMPAR) subunit GluA2 (Harraz et al., 2012), miR-137 participates in the decreased expression of the AMPAR subunit GluA1 (Olde Loohuis et al., 2015) and miR146a-5p participates in controlling the number of AMPARs in synapses as well as synaptic transport processes (Chen and Shen, 2013). These miRNAs influence synaptic plasticity, thereby participating in the regulation of cognitive function.

## 3. Changes of synaptic plasticity in PSCI

Synapses can be regarded as hubs where neurotransmitters are released across the synaptic cleft, and bind to receptors, thereby carrying information from the pre-synaptic neuron to the postsynaptic neuron (Bellot et al., 2014). The strength of synaptic connections and efficiency of synaptic transmission directly affect the processing and storage of information within the central nervous system (Raven et al., 2018).

### 3.1. Changes of structural synaptic plasticity in PSCI

Alterations in structural synaptic plasticity in the early stage are characterized by the swelling of dendrites and loss of spines (Sigler and Murphy, 2010). The indexes of structural synaptic plasticity such as the dendritic branching, spine density, and mushroom-shaped spines have been confirmed decreased in a mouse model of medial prefrontal cortex ischemia-induced cognitive impairment (Sadigh-Eteghad et al., 2018). With prolonged ischemia duration, clinical evidence indicates that a significant reduction in synaptic density is indicated at 21 ± 8 days after stroke onset (Michiels et al., 2022). Chronic lack of blood supply can also lead to the instantaneous loss of synaptic number (Sigler and Murphy, 2010). The decreased number, density, and distribution of synapses reduce the strength of synaptic connections, which are widely regarded as the basis of learning and memory.

### 3.2. Changes of functional synaptic plasticity in PSCI

As for the changes of functional synaptic plasticity, on the one hand, postsynaptic depolarization is caused by the voltage-dependent release of magnesium ions, followed by the rapid permeation of Ca^2+^ ions through the NMDAR to activate Ca^2+^/calmodulin-dependent protein kinase II (CaMKII), resulting in the insertion of AMPARs into the postsynaptic membrane to enhance postsynaptic responses (Malenka and Bear, 2004). The inhibition of ERK and the activation of GABA_A_ receptor after stroke can reduce the postsynaptic depolarization to decrease hippocampal LTP, thus downregulating the efficacy of synaptic transmission. On the other hand, excessive release of glutamate after stroke finally leads to excessive activation of NMDAR, and then the Ca^2+^ move quickly through the NMDAR. After that the protein phosphatases calcineurin and protein phosphatase 1 are activated and their expression levels are upregulated, resulting in the internalization of AMPARs (Kauer and Malenka, 2007). The above changes are called LTD, which means that the prolonged low-frequency stimulation results in the decrease in synaptic efficiency. The low efficiency of synaptic connections affects the brain’s ability to take in and process external information from the outside, eventually manifesting as cognitive impairment.

### 3.3. Changes of synaptic plasticity-related proteins in PSCI

Synaptic plasticity-related proteins refer to those proteins in axon terminals, pre-synaptic membranes, postsynaptic membranes, and synaptic vesicles, include growth-associated protein 43 (GAP-43), postsynaptic density protein 95 (PSD-95), synaptophysin (SYN) and so on. These proteins not only influence synaptic morphology and structure by participating in synaptogenesis and remodeling, but also participate in the function of inter-synaptic information transfer. Participating in long-term plasticity and memory function, GAP-43, a synaptic protein in growth cones, can be regarded as a marker of axonal growth and of morphologic changes in synapses (Baumgärtel and Mansuy, 2012; Nemes et al., 2017). PSD-95, related to receptors and cytoskeletal elements at synapses, increases the number and size of dendritic spines, enhances the maturation of the pre-synaptic terminal, coordinates synaptic maturation, and stabilizes postsynaptic membranes (El-Husseini et al., 2000). As an abundant phosphoprotein present on the membranes of synaptic vesicles, SYN contributes to the development of synapses and increases neurotransmission and spatial memory (Hao et al., 2017). Levels of synaptic plasticity-related proteins GAP-43, PSD-95, SYN have been confirmed to be decreased in a mouse model of PSCI (Sadigh-Eteghad et al., 2018).

## 4. Chinese herbal drugs *via* regulating synaptic plasticity to treat PSCI

Because the pathogenesis of PSCI is determined by various factors and their complex interplay, it is necessary to consider developing therapeutic strategies which target the pathological changes of PSCI. Among these, Chinese herbal drugs, as a class of natural medicinal products based on the theory of traditional Chinese medicine, have great potential to improve synaptic plasticity thus improving cognitive function (Bordet et al., 2017). In our previous research, python language data was used to mine the medication rules of Chinese herbal drugs in the treatment of PSCI and the 38 Chinese herbal drugs that were used more than 10 times were excavated (Shen et al., 2022). After scrutinizing the literature about the mechanism of the above 38 Chinese herbal drugs, we summarized the Chinese herbal drugs that reverse cognitive impairment by improving synaptic plasticity. According to the clinical efficacy of traditional Chinese medicine, we divided twelve Chinese herbal drugs into seven classes:deficiency-tonifying Chinese herbal drugs, blood-activating/stasis-resolving Chinese herbal drugs, sedative Chinese herbal drugs, transforming phlegm and treating cough and asthma Chinese herbal drugs, resuscitation-inducing aromatic Chinese herbal drugs, diaphoretic Chinese herbal drugs and liver-smoothing Chinese herbal drugs. Because of the characteristics of multiple components, the therapeutic mechanisms of in Chinese herbal drugs are complex. The active ingredients of Chinese herbal drugs are not only the material basis of the prescription, but also can be studied to clarify the main mechanism. In order to better elucidate the mechanism and promote the further development and application of the Chinese herbal drugs highlighted above, we have paid particular attention to the active ingredients or extracts.

### 4.1. Deficiency-tonifying Chinese herbal drugs

Deficiency-tonifying Chinese herbal drugs refer to the drugs that can strengthen the body and improve resistance to disease. The commonly used deficiency-tonifying Chinese herbal drugs in treating PSCI include *Epimedium brevicornum Maxim, Herba Cistanches, Panax ginseng C. A. Mey.*, and *Radix Angelica Sinensis*. The common mechanisms involved are promoting the expression of neurotrophic factors, suppressing oxidative stress, and inhibiting cell apoptosis to regulate synaptic plasticity.

#### 4.1.1. *Epimedium brevicornum Maxim*

The natural medicine *Epimedium brevicornum Maxim* mainly contains flavonoids, polysaccharides lignans, alkaloids, and other active ingredient. Among these, icariin is the most useful and active ingredients, which can alleviate vascular cognitive impairment by regulating expression of neurotrophic factors, preventing oxidative stress, inhibiting neuroinflammatory responses, inhibiting apoptosis of nerve cells, and promoting neuronal regeneration (Jiang and Wu, 2020). Previous research showed that the numbers of neurons in the hippocampal CA1 regions of the icariin medium-dose and high-dose groups were higher than those in the model group, indicating that icariin can alleviate neuronal injury and improve structural synaptic plasticity (He et al., 2022). Furthermore, icariin not only increases the levels of acetylcholine and choline acetyltransferase in central cholinergic neural circuits, but also maintains histone acetylation homeostasis, thereby improving cognitive function (Wang, 2013).

#### 4.1.2. *Herba Cistanches*

In classic works of traditional Chinese medicine, *Herba Cistanches* is commonly considered as an effective ingredient to improve intelligence. Among the various chemical components of *Herba Cistanches*, phenylethanoid glycosides represent the most important identified ingredients to uncover and determine the content of *Herba Cistanches* and to improve cognitive function (Rao et al., 2022). As effective ingredients of phenylethanoid glycosides, verbascoside, and echinacoside play different roles in improving cognition. Verbascoside improves memory impairment by reducing oxidative stress, regulating the mTOR signaling pathway, and inhibiting neuronal cell apoptosis (Li et al., 2019). Echinacoside inhibits the activation of microglia cells and astrocytes, reduces inflammatory responses, and releases anti-inflammatory and neurotrophic factors to enhance memory and learning (Wang et al., 2017; Liang et al., 2019). In addition, echinacoside can inhibit the damage induced by over-release of glutamate by reducing voltage-dependent Ca^2+^ entry and suppressing protein kinase (Lu et al., 2016).

#### 4.1.3. *Panax ginseng C. A. Mey.*

Ginsenoside, consisting of ginsenoside Rg1 and ginsenoside Rd are the main active ingredients in *Panax ginseng C. A. Mey.*, which has a variety of neuroprotective effects and causes cognitive improvement after stroke. After a stroke, ginsenoside Rg1 can reduce the NO activity in neurons (He et al., 2014), and downregulate the expression of aquaporin 4 and protease-activated receptor-1 (Zhou et al., 2014; Xie et al., 2015). Moreover, ginsenoside Rd can inhibit the ASK1-JNK pathway and downregulate the expression of caspase-3 (Wang et al., 2014). These two mechanisms may interplay to reduce the damage to neuronal cells. What’s more, ginsenosides can also reduce the damage by free radicals *via* decreased oxidative stress response.

In terms of restoring cognitive function after stroke, accumulating evidence has indicated that Rg1 can also stimulate the differentiation of neural stem cells, increase the secretion of NGF, and induce axonal regeneration (Li et al., 2015). By increasing the expression of vascular endothelial growth factor and BDNF, ginsenoside Rd can activate PI3K-Akt and ERK12 pathways, increase the expression of regulatory transcription factors and genes, and upregulate the expression of GAP-43, thereby improving synaptic plasticity (Liu et al., 2015). Ginsenoside not only plays a role in reducing neuronal cell damage, but also help recover the cognitive function by improving synaptic plasticity.

#### 4.1.4. *Radix Angelica Sinensis*

*Radix Angelica Sinensis* contains the active ingredient Ligustilide, which promotes recovery from cognitive impairment by alleviating neuronal apoptosis and dendritic injury, increasing BDNF and GABA expression to enhance synaptic efficacy (Feng et al., 2012; Xin et al., 2013).

### 4.2. Blood-activating/stasis-resolving Chinese herbal drugs

Blood-activating/stasis-resolving Chinese herbal drugs refer to drugs of which the main effects are promoting blood circulation and dissipating blood stasis. Blood-activating/stasis-resolving Chinese herbal drugs include *Ligusticum chuanxiong Hort, Salvia miltiorrhiza Bunge*, and *Carthamus tinctorius L.*, mainly through reducing oxidative stress and inhibiting apoptosis to regulate synaptic plasticity and thereby improve the cognitive function.

#### 4.2.1. *Ligusticum chuanxiong Hort*

The active ingredients of *Ligusticum chuanxiong Hort* include tetramethylpyrazine, ligustilide, ferulic acid, caffeic acid, and chlorogenic acid. Tetramethylpyrazine, one of its effective active ingredients, has antioxidant, anti-inflammatory and anti-apoptotic properties. It can regulate autophagy and prevent mitochondrial damage to maintain the energy supplement of neurons, thus it plays a neuroprotective role and enhance synaptic plasticity to improve cognition function (Lin et al., 2022). Tetramethylpyrazine can reduce the over-activation of microglia and the NF-κB signaling pathway, down-regulate the level of TNF-α, and inhibit caspase-3 expression, thereby preventing neuronal apoptosis and promoting cognitive recovery (Liang et al., 2022; Zhang et al., 2022). In addition, tetramethylpyrazine could increase BDNF levels, regulate the expression of the synapse-associated proteins PSD-95, SYN, GAP-43, and SYP by activating the TrkB/ERK/CREB signaling pathway (Tan et al., 2021), and increases PSD-93 and PSD-95 expression by restoring the normal function of the cAMP/PKA/CREB pathway. Thus, tetramethylpyrazine directly regulates synaptic plasticity to accelerate the recovery of cognitive function (Wu et al., 2013).

Other ingredients of *Ligusticum chuanxiong Hort* also play an important role in the treatment of neurological diseases. For example, ligustilide can prevent oxidative stress, and regulate endoplasmic reticulum stress and autophagy to reduce neurotoxicity, whereas ferulic acid can inhibit microglial activation, prevent oxidative stress, and reverse mitochondrial dysfunction (Zou et al., 2022). Chlorogenic acid can inhibit increases in NO levels, prevent the release of TNF-α, slow down the breakdown of acetylcholine and butyrylcholine in the brain, improve the activity of the mitochondrial complexes I, IV, and V, reduce the mitochondrial glutathione levels, and modulate Ca^2+^ entry into neurons to protect them from glutamate-induced neurotoxicity (Shen et al., 2012; Mikami and Yamazawa, 2015; Singh et al., 2020). Finally, caffeic acid can prevent oxidative neurodegeneration by inhibiting acetylcholinesterase and cholinesterase activities, thereby slowing down the breakdown of acetylcholine and butanylcholine in the brain (Oboh et al., 2013).

#### 4.2.2. *Salvia miltiorrhiza Bunge*

Tanshinone IIA, the most utilized active ingredient of *Salvia miltiorrhiza Bunge*, is regarded as an effective drug candidate with the broad-spectrum potential for the treatment of neuronal damage and cognitive impairment. Tanshinone IIA attenuates neuronal damage by reducing neuroinflammation and oxidative stress, inhibiting cell apoptosis, recovering blood–brain barrier dysfunctions, and even promoting neurogenesis and angiogenesis (Subedi and Gaire, 2021). Moreover, it can attenuate intracellular Ca^2+^ overload induced by excitatory amino acids, and the impairment of LTP (Wang et al., 2011).

#### 4.2.3. *Carthamus tinctorius L.*

Hydroxy saffron yellow A is the main active component of *Carthamus tinctorius L.* and exerts neuroprotective and cognitive regulatory effects (Xing et al., 2016). Hydroxy saffron yellow A can inhibit the release of inflammatory mediators, reduce free radical responses, exert antioxidant effects, and play the anti-apoptotic effects by regulating the PI3K/Akt signaling pathway (Wang et al., 2022). It also regulates hippocampal synaptic plasticity by enhancing the endogenous expression of VEGF, NR1, BDNF, GluN2A, and GluN2B (Zhang et al., 2014), and improving presynaptic neurotransmitter release and postsynaptic AMPA receptor function (Xing et al., 2016). What’s more, it can restore the damaged LTP amplitude at CA3-CA1 synapses (Yu et al., 2018).

### 4.3. Sedative Chinese herbal drugs

#### 4.3.1. *Polygala tenuifolia Willd.*

In *Polygala tenuifolia Willd.*, the main ingredients that play a role in improving cognitive impairment are triterpenoid saponins, and oligosaccharide esters (Hong et al., 2017). As the most utilized ingredient to improve cognitive impairment, tenuigenin inhibits acetylcholinesterase activity to improve the cholinergic system, exerts antioxidant effects by reducing malondialdehyde levels and increasing superoxide dismutase activity, and enhances field excitatory synaptic potential amplitude to improve synaptic plasticity (Huang et al., 2013). DISS and tenuifoliside (A, B, and C), as the active ingredient of oligosaccharide esters, can inhibit NOS hyper-activation, increase CREB phosphorylation, regulate BDNF expression, promote neuronal cell proliferation, and improve synaptic plasticity (Shi et al., 2013).

### 4.4. Transforming phlegm and treating cough and asthma Chinese herbal drugs

#### 4.4.1. *Ginkgo biloba L.*

The medicinal products derived from the natural medicine *Ginkgo biloba L.* have been widely used to treat neurological diseases. Among them, EGb 761 and ginkgolide have the effect of improving cognitive function. Although it is not a single active ingredient, EGb 761, is the most commonly used extract derived from *Ginkgo biloba L.*, and its main active ingredients are flavonoids (24% flavone glycosides), terpene lactones (6%) terpene lactones, and ginkgolic acid (< 5 ppm) (Nash and Shah, 2015). EGb 761 has been shown to improve mitochondrial function to increase metabolic rates, enhance the connection of neurons in the hippocampus to improve synaptic plasticity, and reduce blood viscosity to ensure the sufficient blood supply to brain regions, thereby alleviating symptoms of cognitive impairment after stroke (Müller et al., 2012).

As an important active ingredient of *Ginkgo biloba L.*, the application of ginkgolide in improving PSCI has attracted increasing attention. Moreover, ginkgolide can reverse oxidative DNA damage in neurons (Hao et al., 2013), promote Bcl-2/Bax expression, which are important oncogenes involved in apoptosis, reduce the expression of activated caspase-3 and decrease intracellular levels of reactive oxygen species, thereby inhibit cell apoptosis and reduce intracellular oxidative stress response (Xia et al., 2014). Ginkgolide could also repair ultrastructure damage of glial cells in the CA1 region of the rat hippocampus, modulate inflammatory responses, support the formation of neurovascular units, nourish neurons and protect synapses (He et al., 2012).

Yinxing Tongzhi tablets are widely used in clinical practice and consist of flavone glycosides and terpene lactones. When combined with western medicine, reports show that Yinxing Tongzhi tablets regulate inflammatory responses by reducing the expression of pro-inflammatory factors such as IL-6 and IL-8, and protect nerve cells by reducing MBP, S100β, and NSE expression (Pan et al., 2018; Yang et al., 2020).

### 4.5. Resuscitation-inducing aromatic Chinese herbal drugs

#### 4.5.1. *Acorus tatarinowii Schott*

α-asarone and β-asarone are regarded as the main ingredients of *Acorus tatarinowii Schott* that improve cognitive function. α-asarone could restore the metabolic imbalance of free radicals which is closely related to the decline in learning and memory function through reducing the MDA levels in hippocampal brain tissue. Moreover, α-asarone inhibits SOD and NOS activity, downregulates the expression of nNOS proteins, and upregulates nNOS/NO signaling in the hippocampus, thereby increasing synaptic plasticity in the hippocampus (Zhu et al., 2020). Evidence indicates that β-asarone may act *via* the Arc/Arg3.1 and Wnt signaling pathways to regulate synaptogenesis, attenuate the spine density reduction in the hippocampal CA1 region, and increase the expression of the synaptic plasticity-related factor GAP-43 in the hippocampus (Yang et al., 2016; Sun et al., 2020). Furthermore, β-asarone can inhibit phosphorylation of the JNK signaling pathway in hippocampal neurons, upregulate Bcl-2 expression, and downregulate caspase-3 expression, thereby playing an anti-apoptotic role in hippocampal neurons. Using Python software to analyze the prescription of traditional Chinese medicine in the treatment of PSCI, *Acorus tatarinowii Schott* was found to be the most clinically applied natural medicine (Shen et al., 2022).

### 4.6. Diaphoretic Chinese herbal drugs

#### 4.6.1. *Radix Puerariae*

Puerarin, a major ingredient of *Radix Puerariae*, reduces the brain infarct size after stroke, attenuates apoptosis *via* activation of the PI3K/Akt signaling pathway (Han et al., 2012), abrogates NMDAR expression after stroke and prevents the toxic effect of excitatory amino acids (Zhang et al., 2011). In addition, puerarin can repair neuronal disorders, restore the synaptic microstructure, reduce neuronal oxidative stress (ROS/SOD/MDA) levels, improve endothelial dysfunction, and inhibit apoptosis by upregulating pro-apoptotic factors (Bax) and downregulating anti-apoptotic factors (Bcl-2) (Zhu et al., 2021).

### 4.7. Liver-smoothing Chinese herbal drugs

#### 4.7.1. *Rhizoma Gastrodiae*

Gastrodin the main active ingredient extracted from *Rhizoma Gastrodiae* has been widely applied in central nervous system disorders, and is a mature, sustainable industrial product used to treat vascular cognitive impairment (Deng et al., 2022). Gastrodin increases the activity of choline acetyltransferase, decreases the activity of acetylcholinesterase and glutamate, and regulates the brain cholinergic system after stroke (Zhang et al., 2008). Gastrodin also exerts antioxidant effects by modulating the total levels of glutathione peroxidase and thiol and attenuates cellular autophagy by inhibiting Ca^2+^/CaMKII signaling (Li and Zhang, 2015; Chen et al., 2021). In addition, the AMPK/UCP2 signaling pathway is activated by gastrodin to improve mitochondrial structure and energy metabolism to ensure sufficient energy supply for synaptic transmission (Sun et al., 2021). In addition, gastrodin directly affects neuronal cells. On the one hand, it can inhibit apoptosis in hippocampal neurons *via* the Nrf2/Keap1-GPx4 signaling pathway (Li et al., 2022). On the other hand, it can induce neural stem cell differentiation by regulating the cAMP/PKA/CREB signaling pathway (Ma et al., 2020). Furthermore, gastrodin can repair axons in the peripheral nervous system and to promote the growth of functional axons and myelin regeneration, and reconstruct the peripheral neural microvascular network (Deng et al., 2022; Yang et al., 2022).

## 5. Conclusion

PSCI refers to a disorder of cognitive dysfunction after the occurrence of a stroke and mainly manifests as an impairment in the five core cognitive domains, including executive function, attention, memory, language ability, and visual-spatial ability (Wang et al., 2021). PSCI highlights the potential causal relationship between stroke and cognitive impairment. The pathological damage of stroke including the increased release of excitatory amino acid, oxidative stress, inflammatory responses, apoptosis, changed neurotrophic factor levels and gene expression influence synaptic plasticity. The changes of synaptic plasticity in PSCI include the changes of structural and functional synaptic plasticity. Structural synaptic plasticity highlights the strength of synaptic connections while functional synaptic plasticity refers to the improvement or inhibition of the efficiency of synaptic transmission. What’s more, synaptic plasticity-related proteins also reflect the changes of synaptic plasticity. Chinese herbal drugs that integrate their active ingredients or extracts with the mechanism of regulating synaptic plasticity have been proven to play a role in improving cognitive impairment. According to the clinical efficacy in traditional Chinese medicine, the 12 most commonly used Chinese herbal drugs can be divided into seven classes. The above seven classes’ drugs both can play anti-oxidative effects to regulate synaptic plasticity. Except for sedative Chinese herbal drugs, the other six classes of drugs can through inhibiting cell apoptosis to regulate synaptic plasticity. Deficiency-tonifying Chinese herbal drugs and sedative Chinese herbal drugs both promote the expression of neurotrophic factors to regulate synaptic plasticity. In addition, *Herba Cistanches, Ligusticum chuanxiong Hort, Salvia miltiorrhiza Bunge and Radix Puerariae* can reduce the damage induced by disturbed glutamate system. *Herba Cistanches, Epimedium brevicornum Maxim, Salvia miltiorrhiza Bunge and Ginkgo biloba L.* regulate inflammatory responses to restore synaptic plasticity, while *Ginkgo biloba L.* can also reverse DNA damage. In summary, Chinese herbal drugs mainly through anti-oxidative stress effect and inhibition of cell apoptosis to regulate synaptic plasticity thereby treating PSCI, which is consistent with evidence shown in previous studies that oxidative stress and neuronal apoptosis are strongly associated with the development of PSCI (Feng et al., 2013; Zhang et al., 2021). Summarizing the mechanism of Chinese herbal drugs in treating PSCI can guide the selection of drugs and the development the novel formulas.

In this review, we overview the influence of pathological damage of stroke on synaptic plasticity, analyze the important roles of synaptic plasticity changes in PSCI, and summarize those Chinese herbal drugs of which the active ingredient or extracts regulate synaptic plasticity to improve cognitive function. We hope that this review will contribute to the summarize the relationship between PSCI and synaptic plasticity, compile evidence of applying Chinese herbal drugs to treat PSCI, and lay a foundation for the development of novel formulas for treating post-stroke cognitive impairment.

## Author contributions

XC conceived and designed the study and drew the figures. LW and HL revised the manuscript. WS and YZ directed the research. All authors contributed to the article and approved the final manuscript.
